# The Impact of 3D Nichoids and Matrix Stiffness on Primary Malignant Mesothelioma Cells

**DOI:** 10.3390/genes15020199

**Published:** 2024-02-01

**Authors:** Stefania Oliveto, Paolo Ritter, Giorgia Deroma, Annarita Miluzio, Chiara Cordiglieri, Mauro Roberto Benvenuti, Luciano Mutti, Manuela Teresa Raimondi, Stefano Biffo

**Affiliations:** 1 Department of Biosciences, University of Milan, 20133 Milan, Italy; oliveto@ingm.org (S.O.); giorgia.deroma@unimi.it (G.D.); 2 National Institute of Molecular Genetics, Fondazione Romeo ed Enrica Invernizzi, INGM, 20122 Milan, Italy; ritter@ingm.org (P.R.); miluzio@ingm.org (A.M.); cordiglieri@ingm.org (C.C.); 3 Department of Chemistry, Materials and Chemical Engineering “Giulio Natta”, Politecnico di Milano, 20133 Milano, Italy; manuela.raimondi@polimi.it; 4 Thoracic Surgery Unit, Department of Medical and Surgical Specialties Radiological Sciences and Public Health, Medical Oncology, University of Brescia, ASST Spedali Civili of Brescia, 25123 Brescia, Italy; mauro.benvenuti@asst-spedalicivili.it; 5 Department of Applied Clinical Sciences and Biotechnology, DISCAB, Aquila University, 67100 L’ Aquila, Italy; luciano.mutti@univaq.it; 6 Department of Biotechnology, SHRO, Temple University, Philadelphia, PA 19122, USA

**Keywords:** NFATC1, epithelioid, organoids, malignant pleural mesothelioma, extracellular matrix

## Abstract

Malignant mesothelioma is a type of cancer that affects the mesothelium. It is an aggressive and deadly form of cancer that is often caused by exposure to asbestos. At the molecular level, it is characterized by a low number of genetic mutations and high heterogeneity among patients. In this work, we analyzed the plasticity of gene expression of primary mesothelial cancer cells by comparing their properties on 2D versus 3D surfaces. First, we derived from primary human samples four independent primary cancer cells. Then, we used Nichoids, which are micro-engineered 3D substrates, as three-dimensional structures. Nichoids limit the dimension of adhering cells during expansion by counteracting cell migration between adjacent units of a substrate with their microarchitecture. Tumor cells grow effectively on Nichoids, where they show enhanced proliferation. We performed RNAseq analyses on all the samples and compared the gene expression pattern of Nichoid-grown tumor cells to that of cells grown in a 2D culture. The PCA analysis showed that 3D samples were more transcriptionally similar compared to the 2D ones. The 3D Nichoids induced a transcriptional remodeling that affected mainly genes involved in extracellular matrix assembly. Among these genes responsible for collagen formation, COL1A1 and COL5A1 exhibited elevated expression, suggesting changes in matrix stiffness. Overall, our data show that primary mesothelioma cells can be effectively expanded in Nichoids and that 3D growth affects the cells’ tensegrity or the mechanical stability of their structure.

## 1. Introduction

Two-dimensional cell cultures are a widely used method for growing cells on flat surfaces, typically made of plastic. The use of 2D culture has been long established and has marked most cell biology studies [1]. However, 2D cultures, in spite of their easiness, present several limitations. First, 2D cultures do not represent the real environment in which cells thrive, lacking the 3D dimension. As a consequence, the use of 2D cultures is not always predictive of several biological phenomena, thus increasing the failure rates of drug discovery. Despite these disadvantages, 2D cell cultures are still used for the majority of cell cultures because they are less expensive than other systems. To overcome the limitations of 2D cultures, several 3D systems have been developed [2,3,4]. In principle, 3D cultures are designed to mimic the natural environment of cells, making them more representative of real cell environments. As such, 3D cultures are used in various fields, such as tissue engineering, drug discovery, and cancer research [5]. Nevertheless, 3D cultures currently lack extensive standardization, with multiple available protocols which do not offer adaptability to all cellular systems [6]. In addition, the effects of 3D cultures on the process of establishing cell lines and on gene expression are rarely studied.

In addition to the 3D environment, the signaling generated by an extracellular matrix (ECM) also has a strong impact on cultured cells. The ECM is a complex network of proteins and carbohydrates that surrounds cells in vivo [7,8]. In a cell culture, the ECM plays a crucial role in cell attachment, proliferation, differentiation, and survival. The ECM provides a physical support for cells to attach to and grow and also serves as a reservoir for growth factors and signaling molecules that regulate cell behavior [9] and translation [10]. Classically, the capability of tumor cells to survive in the absence of ECM is considered one of the golden rules for establishing the status of tumor versus normality during cell culture [11]. In addition to the classical signaling of ECM molecules that relies on the stimulation of specific receptors, such as integrins [12], ECM mechanical properties have attracted novel interest. The stiffness of the matrix refers to the intrinsic rigidity of the surface on which cells are grown. Stiffness is a mechanical property that describes how much force is required to deform the matrix and can be measured in terms of Young’s modulus, which is a measure of a material’s resistance to deformation [13,14]. The relevance of matrix stiffness in a cell culture is significant. The stiffness of the matrix or substrate can influence various cellular functions such as cell migration, spreading, proliferation, phenotype, and differentiation. Moreover, studies have shown that cells respond differently to mechanical cues under 3D microenvironments compared to 2D microenvironments. The stiffness of the matrix or substrate can also affect the morphology of cells, impacting their functionality. Cells seeded on a laminin-rich gel have a significantly less pronounced mechanical response compared to those seeded on gels abundant in collagen and fibronectin [15], suggesting that laminin may be less stiff than fibronectin and collagen. Whether matrix properties and stiffness can be exploited to induce specific phenotypes is an important issue. 

The Nichoid is a micro-engineered substrate used in cell cultures. It is made of microstructures that limit the dimension of the adhering embryoid bodies during expansion by counteracting cell migration between adjacent units of the substrate through its microarchitecture. Nichoids are made up of 25 repetitive niche units, 30 μm high and 90 μm × 90 μm in transverse dimensions, consisting of a lattice of interconnected lines, with a graded spacing between 10 and 30 μm (30, 20, 10, 20, 30) transversely and a uniform spacing of 15 μm vertically. The Nichoid has been shown to maintain the function of pluripotent stem cells when expanded under feeder-free conditions [16]. It has also been found to increase the adhesion and biological expression of stem cells in a way reminiscent of their natural physiological environment. The capability of the Nichoid to maintain stemness may be, in principle, exploited to derive primary tumor cells. In this study, we developed a new strategy for deriving freshly established cell lines from primary tumor samples. Then, we investigated the effects of growing them on 2D surfaces compared to 3D micro-engineered Nichoids. We performed our work on primary tumor cells, derived from four different patients. We showed that 3D Nichoids impart specific clues to tumor cells, inducing, in all lines, a common signature which includes extracellular matrix components. In conclusion, we confirmed that 3D cultures and matrix stiffness are able to steer the fate of tumor cells.

## 2. Materials and Methods

### 2.1. Nichoid Microfabrication

The construction of Nichoids (Moab Srl, Milano, Italy) involves a meticulous fabrication process utilizing two-photon polymerization (2PP). This advanced technique enables the polymerization of a photoresist onto a 12 mm cover glass, generating a three-dimensional lattice structure. The Nichoid architecture comprises a sequence of grids sustained by columns, creating well-defined graded micrometer-scale pores. It consists of 218 square blocks; each block encompasses 5 × 5 structures. These individual units, measuring 90 × 90 × 30 μm^3^, are composed of a multi-tiered arrangement of interconnected rods. In this work, we compared two different geometries, 2D and 3D Nichoids. Two-dimensional Nichoids are flat structures with a grid; 3D Nichoids have vertical walls and are multitiered. Details of fabrication are reported in [16].

### 2.2. Cell and Culture Conditions

Cell lines were maintained following standard tissue culture protocols. Culturing of cells was performed in a humidified environment at 37 °C with 5% CO_2_ using Dulbecco’s Modified Eagle Medium (DMEM) (Gibco, Thermo Fisher Scientific, Monza, Italy, catalog no. 21969-035) supplemented with 10% fetal bovine serum (FBS) (EuroClone, Milan, Italy, catalog no. ECS5000L), 1% penicillin–streptomycin (EuroClone, Milan, Italy, catalog no. ECB3001D), and 2mM L-glutamine (EuroClone, Milan, Italy, catalog no. ECB3000D). We resuspended cells to a final concentration of 500/µL in culture medium [17]. To guarantee a uniform cell seeding inside the Nichoid, we first plated cells in 100 μL volume and, after one hour, we added 2 ml of complete medium.

### 2.3. Primary Tumor Cells Isolation

Primary cells were obtained from freshly resected surgical specimens. Informed consent was obtained from all patients and the study received approval from the Ethics Committee of Bari CT0523978 on 11 November 2021. The patients, all male and aged between 70 and 75 years, had a history of past asbestos exposure. Histopathological analysis confirmed that all collected samples were classified as the epithelioid subtype. None of the patients had undergone neo-adjuvant chemotherapy and/or radiotherapy. In brief, tumor tissue samples were placed in a sterile dish, cleaned with PBS 1X (EuroClone, Milan, Italy, catalog no. ECB4004L), and cut into smaller pieces. Pieces were then transferred to a sterile flask and digested to single cells using a mixture of collagenase I-II-IV (1 mg/mL each, Gibco, Thermo Scientific, Monza, Italy: Collagenase type I, catalog no. 17018-029; Collagenase type II, catalog no. 17101-15; Collagenase type IV, catalog no. 17104-019) for 4 h at 37 °C. The resulting cell suspension was filtered through a cell strainer to remove undigested tissue fragments and then centrifuged at 300 g for 10 min to pellet the dissociated cells. The cell pellet was resuspended in culture medium, plated in a flat condition, and maintained for up to 2 weeks.

### 2.4. Nichoid Support Generation

We needed to confine cells within the surface covered by the polymerized structures of the Nichoid and avoid seeding on the surrounding structures. For this purpose, we mounted Nichoids on the top of ultra-low attachment 6-well plates (Costar, Corning Incorporated, Tewksbury MA, USA, catalog no. 31223006). To eliminate the glass annulus that remained around the polymerized Nichoid post-development, holes with a diameter of 7 mm were created at the bottoms of the wells using a lathe. The samples were affixed to the external part of the well using a biocompatible Loctite AA 3321 glue (Henkel, Milan, Italy), which polymerized under a UV lamp with a wavelength of 365 nm (Hamamatsu Photonics, Roma, Italy). For sterilization, wells underwent a thorough cleaning process. They were washed with sterile deionized water, cleansed with 70% ethanol for at least 30 min, rinsed again with deionized sterile water, dried, and left overnight (o/n) under the UV lamp within a sterile hood.

### 2.5. Proliferation Assay

For the proliferation assay, the CellTrace™ Far Red Cell Proliferation Kit (Thermo Fisher Scientific, Monza, Italy, catalog no. C34572) was employed. A total of 1 μL of CellTrace^TM^ stock solution was added to each ml of cell suspension and incubated for 20 min at 37 °C, shielded from light. After washing to remove any free dye remaining in the solution, the cells were pelleted, resuspended in fresh complete culture medium, and then seeded. 

### 2.6. Immunofluorescence and Imaging

Phalloidin (Sigma-Aldrich, St. Louis, Missouri, USA, catalog no. P5282) and anti-nucleophosmin (Abcam, Cambridge, UK, catalog no. ab15440) were used to stain, respectively, cytoplasmic microfilaments and nucleoli, as previously described [18]. Briefly, after permeabilization with 0.5% Triton-X 100 in PBS, cells were incubated with primary antibodies, washed, and the reaction revealed with fluorescent secondary antibodies (Thermo Fisher Scientific, Monza, Italy, catalog no A-11008). Instrument used and configuration: Nikon-Crest multimodal spinning disk, with 4-laser (LDI, Ltd. Instruments, Tallinn, Estonia) and 16-LED excitation (Pe-Cool LED), equipped with Andor EM-CCD and Andor sCMOS cameras, used here in widefield modality (pinhole aperture 180 micron), for both differential interference contrast imaging (DIC) and fluorescence imaging of both far-red emission (excitation LED line at 635 nm; emission filter 680/20 nm) and green emission (excitation LED line at 490 nm; emission filter 530/20 nm), plus blue emission for scaffold matrix reflection (excitation LED line at 385nm; emission filter 415/20 nm), using 4× and 10× air objectives (all from Nikon instruments). Three-dimensional images were acquired over 30um Z stacks (5 Zstep, with Z-distance 12.5 um). Images were processed in order to suppress the background and increase the signal-to-noise ratio both in scaffold grid and cellular signals.

### 2.7. RNA Collection and Sequencing

Total RNA was isolated using the TRIzol reagent (Total RNA Isolation reagent, Invitrogen, Thermo Fisher Scientific, Monza, Italy, catalog no. 15596026) following the standard protocol. The extracted RNA was quantified with the Qubit 2.0 Fluorometer (Invitrogen, Thermo Fisher Scientific, Monza, Italy), and its quality and integrity were evaluated through the Agilent 2100 Bioanalyzer, the Agilent RNA 6000 Pico Kit, and the RNA Pico Chips (Agilent Technology, Santa Clara, California, USA, catalog no. 5067-1513). Four biological replicates have been prepared for the sequencing. Each of the samples contained a total of 100 ng of RNA [19]. A single-end (1 × 75) run was performed on an Illumina HiSeq 2500 Sequencing System (IGA Technologies Services, Udine, Italy) with a requirement of 50 million reads per sample. The RNA was fragmented and reverse-transcribed into cDNA; random primed cDNA libraries were constructed using the Universal Plus mRNA-Seq kit (Tecan Genomics, Redwood City, CA, USA, catalog no. 0520-24). FastQ files of the reads were generated. Raw reads were then subjected to a quality control by FastQC software (version 0.11.8) and filtered by Trimmomatic (version 0.39). Raw data were mapped onto a reference genome using STAR software (v.2.5.0); then, read counts for each detected gene were obtained using HTSeq-Counts algorithm (version 0.11.1) with default parameters (gene annotation release 98 from Ensembl). Finally, the read counts matrix generated by HTSeq-Counts was analyzed by means of the DESeq2 R/Bioconductor package (version 1.24.0) [20]. The following comparison was analyzed: cells cultured in the 3D Nichoids versus cells cultured in the 2D controls. Analyses were performed in R version 3.5.1 [21]. 

### 2.8. Functional Analysis

Gene Ontology enrichment analysis was performed using topGO R Bioconductor package (version topGO_2.24.0), as described in [22]. The annFUN.db function was used to extract the gene-to-GO mappings from the genome-wide annotation library org.Hs.eg.db for H. Sapiens. An additive functional analysis was performed on shinyGO [23]. 

### 2.9. Venn Diagram Analysis

Venn diagrams were obtained according to [24]. Only protein coding genes were used for each condition. In the analysis of common genes, we excluded genes with read count = 0 in at least one of the considered samples. The number of genes considered in this work has been highlighted in the red box.

### 2.10. RT-qPCR

Total RNA was extracted as described in Section 2.7. Next, 1 μg of total RNA was reverse-transcribed using random primers and the SuperScript III First-Strand Synthesis SuperMix (Invitrogen, Thermo Fisher Scientific, Monza, Italy, catalog no. 18080-400). The resulting cDNA was subsequently processed for real-time PCR amplification using Sybr Green Technique and utilizing GoTaq qPCR Master Mix (Promega, Milan, Italy, catalog no. A6001) on a StepOnePlus system (Thermo Fisher Scientific, Monza, Italy). The following primers were used to amplify selected genes: GAPDH (Fwd: 5′-ATGACCCCTTCATTGACC-3′; Rev: 5′-GAAGATGGTGATGGGATTTC-3′), COL1A1 (Fwd: 5′-CTGGACCTAAAGGTGCTGCT-3′; Rev: 5′-GCTCCAGCCTCTCCATCTT-3′), and THBS2 (Fwd: 5′-TCTGAGCAAGTGTGACACC-3′; Rev: 5′–TGCAATTCTTGCCCCCCATC-3′). GAPDH gene was used as an internal standard to normalize samples by ΔΔC_t_ method.

### 2.11. Statistical Analysis

Each experiment was repeated at least three times, as biological replicates; means and SDs between different experiments were calculated. Statistical *p* values obtained by two-tailed Student *t* test were indicated: **, *p* < 0.01; *, *p* < 0.05.

## 3. Results

### 3.1. The Nichoid Scaffold: A 3D Architectural Framework

**Figure 1:**
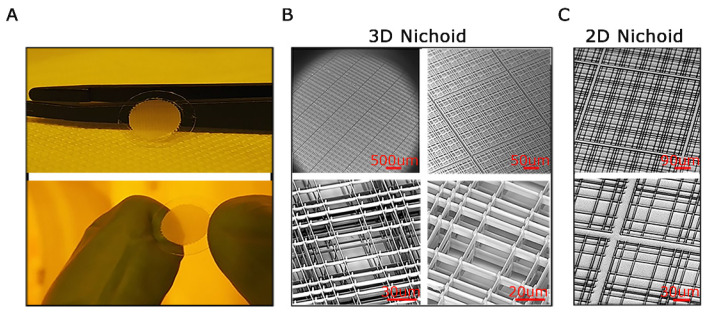
The Nichoid scaffold. (**A**). Pictures of freshly fabricated and developed 3D Nichoid samples; tweezers and fingers for scale reference. (**B**). Scanning Electron Microscopy (SEM) images of 3D Nichoid samples with closeups on hierarchical elements: matrix of blocks of 5 × 5 niches (top left), block of 5 × 5 niches (top right), and single niches (bottom). (**C**). SEM images of the flat patterns of 2D Nichoid samples with closeups on hierarchical elements: a 5 × 5 block (top) and two single niches (bottom). Scale bars are shown in red color.

Three-dimensional scaffolds mimic cellular microenvironments and can be tailored to support specific cell types. The construction process involves fabrication techniques to generate structures that allow cellular interactions, promote cell growth, and enable the study of cellular behavior in a three-dimensional context. In this work, we examined two types of Nichoids: one with a flat design, called 2D Nichoid and another with a layered structure, named 3D Nichoid. The Nichoid scaffold is crafted using a technique called 2PP fabrication, enabling the polymerization of a photoresist on a 12 mm cover glass (Figure 1A). This process results in a structured framework composed of grids connected by columns, creating sized pores at the micrometer scale within the scaffold’s three-dimensional structure. In particular, as shown in Figure 1B the 3D model followed the classic Nichoid design [25], comprising 218 square blocks, each containing 5 × 5 structures. These structures, regarded as elementary units, were sized at 90 × 90 × 30 μm^3^ and contained three levels of interconnected rods. The gaps between the rods in the horizontal direction differed, measuring 10, 20, and 30 μm, respectively [16]. The 2D Nichoid shared the same grid structure, yet it consisted of only a single floor, resulting in a thinner scaffold of 2 μm overall thickness. This resulted in a two-dimensional framework (Figure 1C). We used, therefore, 2D Nichoids as a control of 3D Nichoids.

### 3.2. Isolation and Establishment of Primary Malignant Pleural Mesothelioma Cells

**Figure 2:**
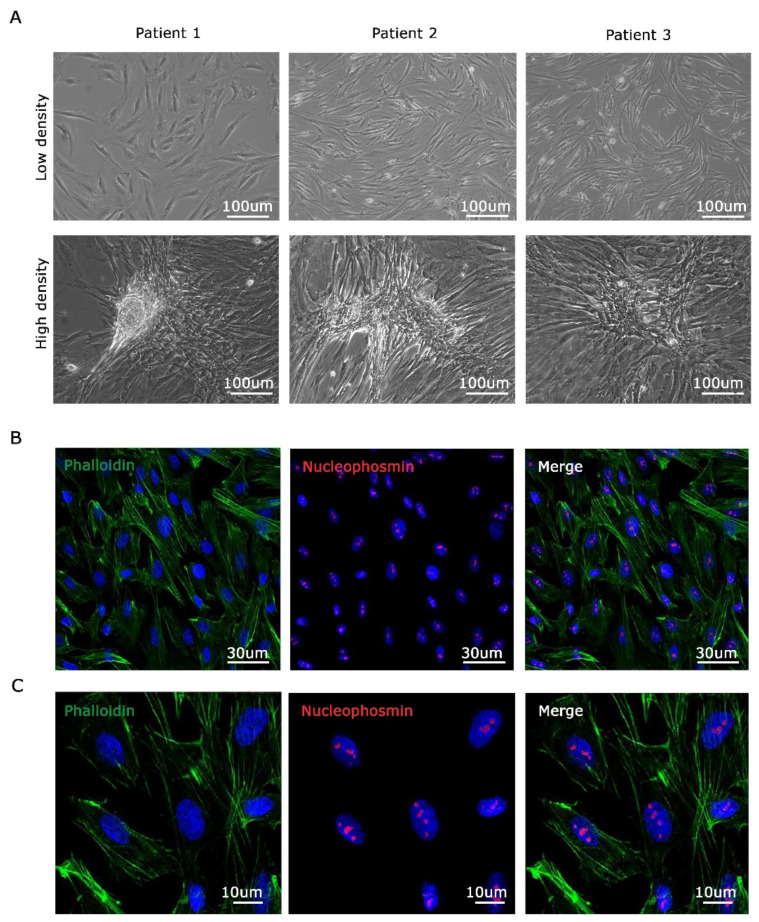
Three MPM cell lines established by isolating cells from biopsies belonging to different patients. (**A**). Images of three different mesothelioma-derived cell lines cultured at low density (top) and high density (bottom). (**B**). Immunofluorescence analysis of derived cells stained with Phalloidin for cytoplasmic microfilaments (green signal), nucleophosmin for nucleolar structure (red signal), and DAPI for nuclei (blue signal). (**C**) High magnification of (**B**). Scale bars are shown.

Malignant mesothelioma, a rare yet aggressive cancer, is often diagnosed in advanced stages, limiting the availability of early-stage tissue samples. In this study, primary cells of malignant pleural mesothelioma (MPM) were generated from surgically resected tissues obtained from patients with epithelioid MPM who had not undergone any prior therapies. These MPM cell cultures were utilized in experiments during their early passages. As shown in Figure 2A, the primary MPM cell cultures were established in a monolayer configuration, displaying a characteristic epithelial-like, cobble-stone appearance, at low density (Figure 2A top). When cultured at high density, all derived cells display increased cell–cell contacts and the formation of multilayered cells structures (Figure 2A bottom). Each cell line exhibits distinct morphology. Finally, to better characterize MPM primary cells, we seeded them at medium density in a flat condition. We then investigated their shape and morphology by staining with phalloidin for actin microfilaments and nucleophosmin for nucleoli [26]. As shown in Figure 2B (low magnification) and 2C (high magnification), primary MPM cells exhibit a well-defined microfilament structure and distinct nucleoli.

### 3.3. MPM Cells have Enhanced Proliferation in the 3D Nichoid Compared to the One-Floor Nichoid

**Figure 3:**
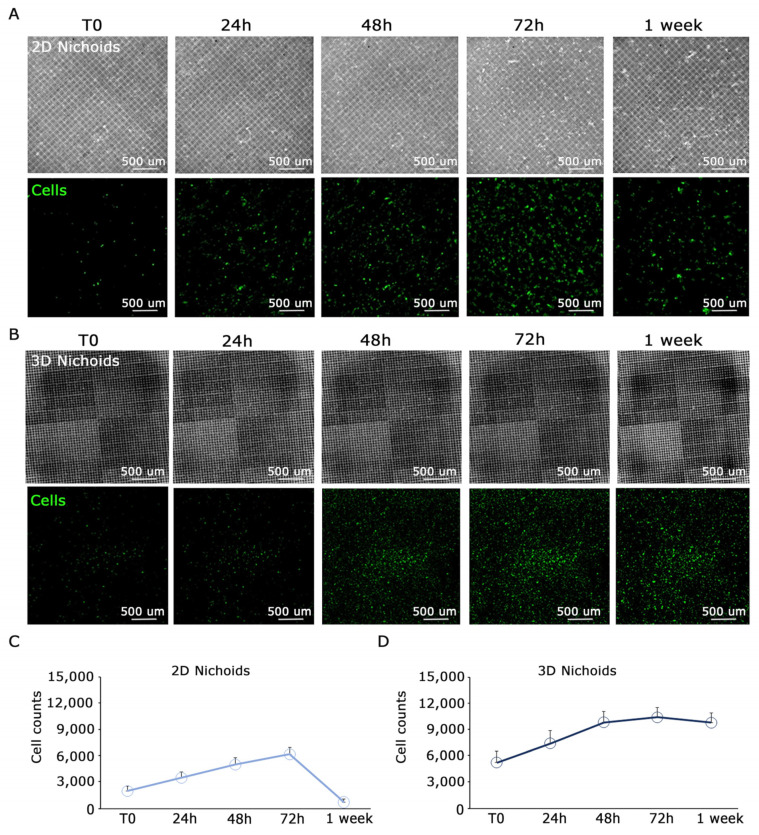
Proliferation analysis of cells in 3D and 2D Nichoid shows a decrease in proliferation at day 3 in 2D condition. (**A**). Top: images of the 2D Nichoid captured in widefield mode at different time points; bottom: images of representative MPM primary cells detached from the Nichoid structure at different time points. (**B**). Top: images of the 3D Nichoid captured in widefield mode at different time points; bottom: images of representative MPM primary cells detached from the Nichoid structure at different time points. In the cells channel, the contrast was increased to emphasize the signal/noise ratio. (**C**,**D**). Plot of cell counts performed on the entire well in 2D Nichoid (**C**) and 3D Nichoid (**D**). After day 3, a considerable decrease in proliferation is observed in the one-floor control, while, in the 3D condition, a plateau is reached. Scale bars are displayed.

**Figure 4:**
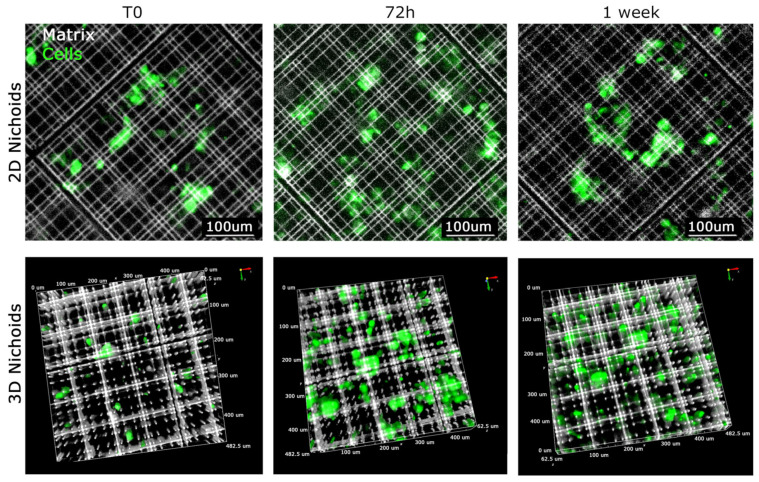
Growth of cells in XYZ dimension in 2D and 3D Nichoid. Visualization of representative primary MPM cells grown in 2D Nichoid (**top**) and 3D Nichoid (**bottom**) at defined time points. The 2D structure is represented by only 1 plan and 3D structure by Z-stack spanning 30 um in thickness. MPM cells stained with CFSE are visualized in green, whereas the Nichoid grids, fluorescence emitting upon violet light excitation (385 nm), are displayed in white. Scale bars and 3D orientation as displayed.

Primary malignant pleural mesothelioma (MPM) cells typically undergo expansion in conventional flat culture substrates. Recognizing the importance of replicating the native three-dimensional tumor environment, our investigation focused on elucidating the behavior of MPM-derived cells cultivated within three-dimensional Nichoids. To establish meaningful comparisons, we incorporated one-floor Nichoids as planar controls, ensuring that any observed distinctions stem solely from differences in dimensionality while keeping cells in contact with the same polymerized resin. Considering the reduction in viability of tumor cells during extended ex vivo culture within a two-dimensional setting, we sought to explore potential differences in proliferation between two-dimensional and three-dimensional settings. Primary MPM cells were stained with CellTrace™ Far Red and seeded in either 3D or 2D Nichoids. Initially, 50,000 MPM cells were drop-seeded onto either one 3D Nichoid or one 2D Nichoid. After allowing cells to adhere for 1 h, culture medium was added to provide essential nutrients. Proliferation was monitored up to day 7, with images captured at each time point using a Nikon-Crest in widefield mode (Figure 3A,B). After signal processing, in order to minimize background interference and enhance signal-to-noise ratio in the far-red cell channel, we generated a mask for cell counting. Cell counts were performed on different areas of the samples, dividing the well into four parts and plotting values (Figure 3C,D). Our findings indicate that cells in the 3D Nichoid exhibit enhanced viability compared to those in the one-floor Nichoid, particularly noticeable from day 3 onward. Cells in the flat condition exhibited a decline in number after day 3 (Figure 3C), whereas those in the three-dimensional setting remained constant (Figure 3D). Furthermore, as illustrated in Figure 4, high-magnification images of CFSE-stained cells underscore the impact of three-dimensionality on cell cultures. The CFSE distribution reveals a flattened cell arrangement in 2D Nichoids (Figure 4, top), resembling traditional adherent attachment. In contrast, in 3D Nichoids, the distribution spans multiple planes (Figure 4, bottom). These morphological features persist over the culture duration in both 2D and 3D Nichoids.

### 3.4. Primary MPM Cells Cultured in 2D and 3D Nichoids Are Characterized by a Different Transcriptional Signature

**Figure 5:**
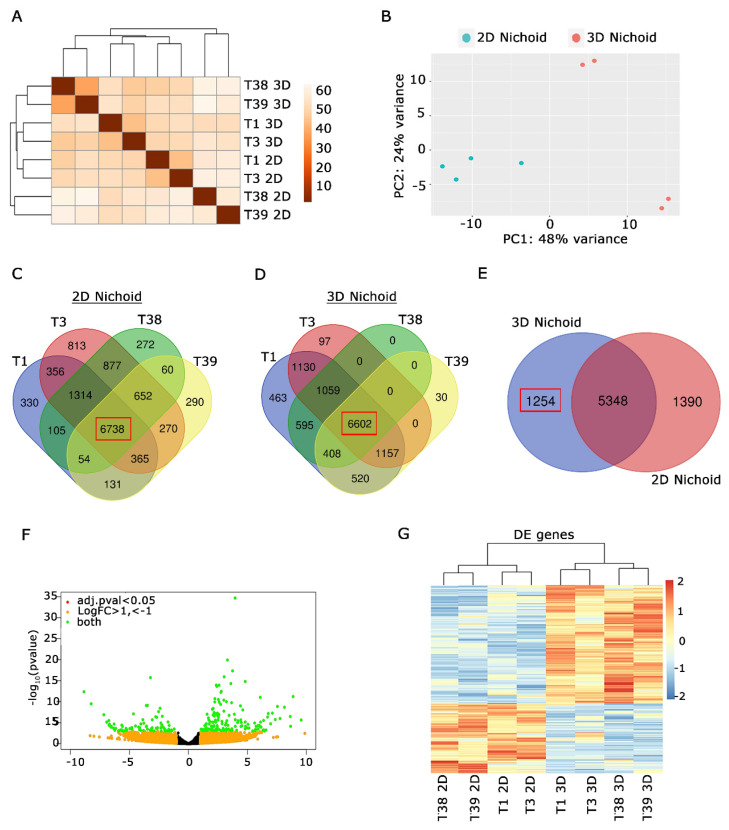
Transcriptional analysis of MPM cells cultured in 2D and 3D Nichoids highlights distinct transcriptional signatures on the three-dimensional culture. (**A**). Distance matrix analysis evidenced the presence of specific clusters among 2D and 3D Nichoids. (**B**). PCA analysis showed that 3D Nichoids are more similar than 2D Nichoids. (**C**) Venn diagram of protein coding genes expressed by 4 primary MPM cells grown in 2D Nichoids. Number of common genes in all possible conditions are indicated. Common genes among all the cell lines are bolded in a red box. (**D**) Venn diagram of protein coding genes expressed by indicated MPM cells cultured in 3D Nichoids. Number of common genes in all possible conditions are indicated. Common genes among all the cells are bolded in a red box. (**E**) Venn diagram obtained by comparing common genes of all indicated cell lines in C and D. Number of protein coding genes specifically expressed in 3D Nichoids is highlighted in the red box. (**F**). Volcano plot evidencing significative deregulated genes among 2D and 3D Nichoids, highlighted in green. Criteria for selection are evidenced: green for |log2(FoldChange)| > 1 and adjusted *p* value ≤ 0.05; orange for log2(FoldChange) <−1 e > 1; red for adjusted *p* value ≤ 0.05. (**G**). Heatmap of significative DE genes among 3D Nichoids vs. 2D Nichoids. Each row represents a gene, while columns depict different samples. The color gradient represents gene expression levels, with red indicating upregulated genes and blue representing downregulated genes in 3D Nichoids compared to 2D Nichoids. Higher color intensity reflects higher expression levels. The analysis highlights distinct transcriptional signatures providing insights into gene expression alterations in response to three-dimensional culture conditions. All analyses were performed in biological replicates using four distinct samples.

To explore the impact of Nichoid’s three-dimensional structure on MPM cells’ gene expression, a comprehensive transcriptome analysis was conducted on Nichoids cultures. We sequenced the RNA of four primary MPM cells (T1, T3, T38, and T39 cells) seeded both in 3D Nichoid (named in figures T*3D) and one-floor Nichoid (named in figures T*2D). A first analysis revealed distinct clusters within the dataset, indicating groups of samples with similar expression patterns (Figure 5A). Also, PCA analysis provided a map of patterns and relationships between 3D and 2D Nichoid samples, evidencing that 3D samples were more transcriptionally similar compared to the 2D ones (Figure 5B). Then, we looked at genes that were specifically expressed across all primary cells, aiming to discern if a gene signature can delineate their behavior. We found that the 3D Nichoid structure enhances gene similarity among primary mesothelioma cells, as depicted in Figure 5C,D. A closer examination of shared genes across different cell lines in the 2D Nichoid revealed genes that were expressed exclusively in one cell line but not in the others (Figure 5C). In contrast, we found a reduced number of genes specifically expressed by each cell line in 3D Nichoids. Notably, the number of unique genes for T38 was null (Figure 5D). 

The majority of genes were shared among all cells in both 2D and 3D Nichoids, as highlighted by red boxes in Figure 5C,D (2D Nichoids: 6738; 3D Nichoids: 6602). We analyzed the overlap of the 6738 2D Nichoid genes with the 6602 3D Nichoid genes. We found 1254 genes expressed exclusively in 3D Nichoid, 1390 in 2D Nichoid, and 5348 genes in both conditions (Figure 5E). The analysis of genes expressed exclusively in 3D Nichoid revealed genes involved in the matrix cellular compartment. To delve more deeply into the transcriptional reshaping of MPM cells, we examined the differentially expressed genes between 2D and 3D Nichoids.

The profiling of the complete transcriptome of Nichoid cultures evidenced that gene expression was affected in a significant way by 3D Nichoids. Indeed, 143 genes were upregulated and 84 downregulated by |log2(FoldChange)| > 1 and adjusted *p* value ≤ 0.05. All these differentially expressed (DE) genes are portrayed in green. By contrast, genes located closer to the center of the plot exhibited lower significance and minimal fold change (Figure 5F). We have identified a subset of genes that were markedly upregulated or downregulated, providing insights into potential biological processes or pathways that may be implicated in the observed experimental conditions. Finally, the representation by color gradient of the DE genes in the heatmap graph (Figure 5G) highlighted the similarities and differences in gene expression profiles among 3D and 2D samples. All together, we conclude that the 3D Nichoid culture induces a specific gene signature remodeling.

### 3.5. Functional Analysis of DE Genes Unravel Extracellular Matrix Involvement

**Figure 6:**
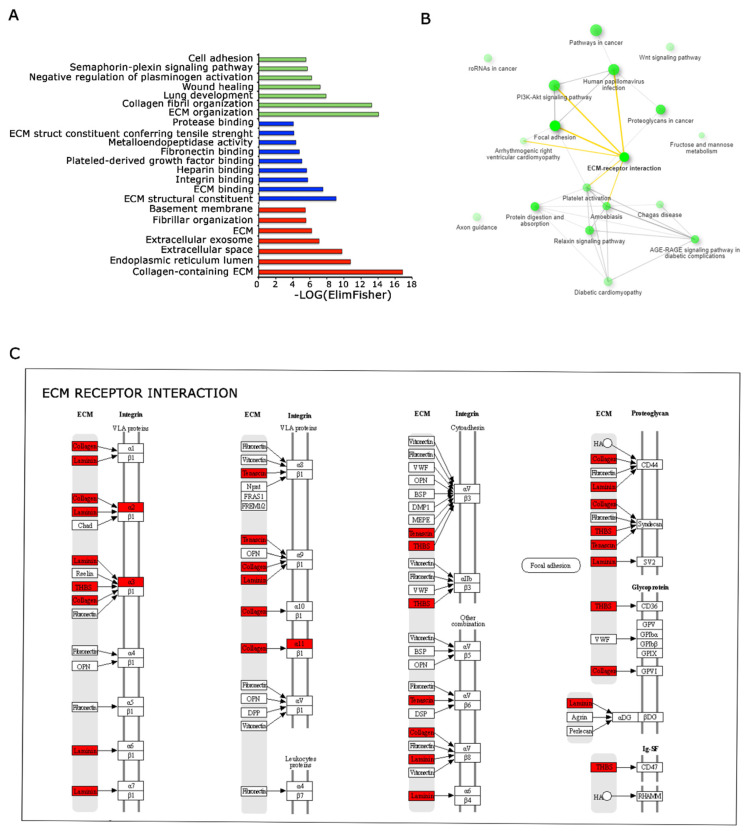
Functional analysis of DE genes among 3D and 2D Nichoids depict the perturbation of the ECM pathway. (**A**). Representation of enriched biological processes (represented in green), molecular functions (represented in blue) and cellular components (represented in red)identified through Gene Ontology analysis. Significantly enriched terms are highlighted, indicating biological pathways implicated in the studied conditions. (**B**). Pathway relationships depicted based on gene enrichment analysis. Interconnected pathways share common genes, emphasizing the interplay and connectivity among molecular pathways influencing cellular responses in the investigated system. ECM interaction is evidenced in bold and links in yellow. (**C**). Pathview representation displaying the ECM interactions enriched with deregulated genes between 3D and 2D Nichoids. Genes highlighted in red represent the upregulated genes in our dataset, emphasizing their involvement in the ECM pathways and cellular interactions.

In order to define and describe more in detail the gene signatures specific for 3D Nichoids, we performed an overrepresentation study. The Gene Ontology (GO) analysis [22] revealed substantial enrichment in biological processes associated with the extracellular matrix (ECM) and cell adhesion, for molecular functions, biological processes, and cellular components. The overrepresentation of terms related to ECM organization indicates an active involvement in structural maintenance, remodeling, and regulation within the ECM. Furthermore, there was a noteworthy enrichment in terms linked to cell adhesion processes, emphasizing the importance of cell–cell and cell–ECM interactions (Figure 6A). Also, network analysis performed with ShinyGO [23] tool confirmed the involvement and the importance of extracellular matrix remodeling, as shown in yellow (Figure 6B). In this analysis, two pathways are connected if they share at least 20% of genes. These results denote a comprehensive engagement of cellular components and molecular pathways governing the ECM and cell adhesion, potentially influencing critical cellular functions and signaling cascades. We also performed iRegulon [27] analysis; NFATC1 ranked first for regulated targets, whereas FOXN1 had the highest enrichment signal.

### 3.6. Genes Deregulated in 3D Nichoids Are Involved in the Cytoskeletal Remodeling

**Figure 7:**
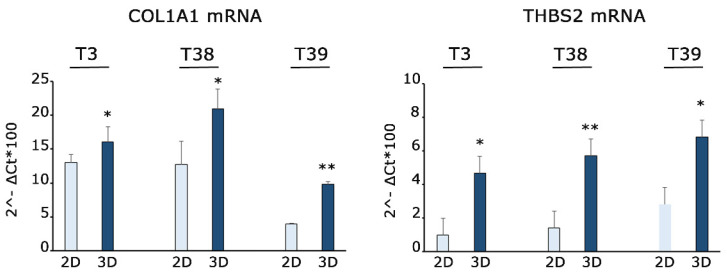
Gene expression quantification shows upregulation of COL1A1 and THBS2 in 3D Nichoids. RT-qPCR for COL1A1 and THBS2 mRNAs performed on three independent primary MPM cells grown in 2D and 3D Nichoids for 4 days. Histograms represent the means ± the SD of three independent experiments. Statistical *p* values were calculated using double-tailed unpaired *t* test. * = *p* < 0.05, ** = *p* < 0.01.

We conducted an in silico analysis, scanning ECM receptor interaction pathways by KEGG tool (Figure 6C). The RNA sequencing results revealed a notable upregulation in the expression levels of several genes in the studied samples. Among genes responsible for collagen formation, COL1A1 and COL5A1 exhibited elevated expression (log_2_FC = 2.361 e 2.404, respectively), suggesting an intensified collagen synthesis process. Notably, the upregulation of Keratin 18 (KRT18) gene expression (log_2_FC = 8.875) indicated a potential shift in cytoskeletal dynamics. Furthermore, the increased expression of Thrombospondin 2 (THBS2, log_2_FC = 2.602) and Laminin Subunit γ 2 (LAMC2, log_2_FC = 5.908) highlighted their possible involvement in cell–matrix interactions and structural modifications. Importantly, the analysis through RT-qPCR of three independent samples confirmed the upregulation of COL1A1 and THBS2 genes in the 3D Nichoids, as displayed in Figure 7. The upregulation of genes related to extracellular matrix components (such as COL1A1 and COL5A1), cytoskeletal elements (like KRT18), and factors involved in cell–matrix interactions (THBS2 and LAMC2) suggests an active induction of matrix remodeling at the transcriptional level induced by 3D Nichoids.

## 4. Discussion

We have demonstrated the derivation and cultivation of tumor cells in 3D Nichoids. These cells, when plated on 3D Nichoids, exhibit viability and express specific genes. Importantly, our study included a comparison between the effects of growing cells on 3D Nichoids and cells growing on 2D Nichoids that had the same surface and chemical composition of 3D Nichoids. This approach allowed us to precisely define the instructive effects of 3D versus 2D structure, thus eliminating the bias generated by the substrate. Furthermore, our experiments were conducted using primary tumor cells. The identified gene signature indicates that 3D Nichoids induce extracellular matrix (ECM) remodeling. The significance of these findings will be discussed in the context of the key role of ECM in pathological processes and cancer progression [28]. 

The Nichoid is a micro-fabricated lattice substrate produced using the two-photon polymerization technique [29]. It is composed of an inert, biocompatible, and mechanically stable photoresin and exhibits highly controlled spatial resolution. Each scaffold beam has a thickness of 1.5 μm, and the Nichoid boasts a porosity of 90%. With pore sizes ranging from 10 to 30 μm in the xy-plane and 15 μm along the z-direction, cells can freely adhere in three dimensions. In comparison to standard glass coverslips, the Nichoid reduces cell adhesion, affecting both the number and maturation of focal adhesions. Numerous studies have explored the effects of the Nichoid in cell culture, revealing a general ability to maintain stem cell properties across various types of stem cells when compared to cells cultivated on 2D surfaces. Initially, it was hypothesized that Nichoid microstructures induce genetic reprograming primarily by controlling cytoskeletal tension [30]. Subsequent research demonstrated that mesenchymal stem cells cultured in the 3D Nichoid exhibited a comparable proliferation rate to flat substrates but were spatially organized in 3D, with smaller and spherical nuclei. In these cells, the differential localization of YAP, a mechanotransducer [31], suggested cytoskeletal reshaping and stiffness-related signaling. In spite of this, gene expression analysis primarily revealed upregulation of genes related to stemness [16] rather than to cytoskeletal changes. More recently, gene expression analysis of mesenchymal stem cells grown on 3D Nichoids demonstrated the dysregulation of 1843 genes, including some ECM components [32]. However, a limitation of previous studies was the use of standard cell culture reagents with different chemical compositions as controls for 2D surfaces, coupled to the analysis of a single cell line. In our work, we utilized four different biological samples freshly derived from surgeries. Additionally, we employed 2D surfaces with the same chemical composition as 3D Nichoids as controls. In this context, we observed a remarkably consistent gene expression remodeling induced by 3D structures. Indeed, we found an unequivocal reshaping of extracellular matrix gene expression. We conclude that the gene expression patterns of cells differing for 3D versus 2D growth show a pronounced cytoskeletal response. In addition, four biologically different samples converge into a consistent gene expression response.

Malignant mesothelioma is a type of cancer that affects the mesothelium, a simple squamous epithelium that lines several cavities, including the pleura around the lungs, the peritoneum, and the pericardium around the heart. The primary risk factor for mesothelioma is asbestos exposure [33]. Despite having a relatively low mutational burden, with a median of 23 mutations per biopsy specimen and approximately 1.2 mutations per Mb [34], malignant mesothelioma is highly lethal [35]. In previous studies, we demonstrated that translational control of gene expression plays a crucial role in mesothelioma progression, either directly through eIF6 [36] or indirectly through microRNA association with polysomes [17]. These findings suggest that the malignancy of mesothelioma cells is linked to post-transcriptional regulation and the ability to establish a supportive microenvironment, minimizing the impact of chemotherapy [37]. In this context, eIF6 was isolated as an integrin binding protein [38], and recent evidence has demonstrated a novel role of eIF6 in mechanical responses of cells [39].

Notably, our experiments, conducted on four biologically diverse samples from different patients, revealed a common transcriptional landscape. Iregulon [27] analysis allowed the identification of transcription factor activities underlying gene expression changes. NFATC1 emerged as the most important transcription factor with stimulated activity on 3D structures. In osteoclasts, NFATC1 plays a crucial role in regulating the expression of osteoclast-specific genes. The short isoform of NFATC1 is essential for osteoclastogenesis and is responsible for the expression of various osteoclast markers, including NFATC1 regulators [40]. Cases of diffuse biphasic malignant mesothelioma with osseous differentiation and long survival have been observed in clinical practice [41]. These observations suggest that the 3D Nichoid, although preserving the viability of mesothelioma cells, may induce differentiating, less aggressive phenotypes. Overall, these data stress the possibility that occult cancer can be controlled by the features of the microenvironment, as suggested long ago [42]. It will be interesting to further study the modalities by which the 3D Nichoid instructs such a change.

The changes in gene expression were confirmed in two different experiments. We selected for validation COLA1A1 and THBS2. COL1A1 in MPM is significantly correlated to the infiltration levels of CD4^+^ T cells, macrophages, and neutrophils [43] and significantly upregulated in hepatocellular carcinoma tumor tissues in comparison to normal tissues [44]. The THBS2 gene encodes for a thrombospondin family protein that mediates cell-to-cell and cell-to-matrix interactions [45]. In short, the changes induced by 3D structures may deeply affect the extracellular environment, an observation accompanied by the fact that mesothelioma cells at high density reach a plateau rather than the loss of viability observed in 2D structures. 

## 5. Conclusions

A novel method for cultivating tumor cells, known as 3D Nichoids, is introduced. Three-dimensional Nichoids have the capability to induce specific transcriptional changes, leading to the remodeling of extracellular matrix proteins. This observation suggests the existence of a crosstalk between 3D shape and gene expression. Although the physiological relevance of these changes is yet to be defined, it is important to note that cellular viability remains uncompromised.

## Data Availability

The data presented in this study are available on request from the corresponding author. The RNAseq data are in Supplementary Table S1. The raw data are available in www.ebi.ac.uk/arrayexpress accessed on 31 March 2024. Accession number E-MTAB-13749.

## Supplementary Materials

The following supporting information can be downloaded at https://www.mdpi.com/article/10.3390/genes15020199/s1. Table S1: Differentially expressed genes among 3D and 2D Nichoids.
