# Supplementary material for: The Impact of 3D Nichoids and Matrix Stiffness on Primary Malignant Mesothelioma Cells
Source: Genes (Basel). 2024 Feb 1;15(2):199. doi: 10.3390/genes15020199 (PMC10887956; doi:10.3390/genes15020199)
Supplement: Supplementary file 1 [file genes-15-00199-s001.zip › genes-2816146-supplementary.pdf]

ensembl_gene_id	external_gene_name	log2FoldChange	pvalue	padj	description	gene_biotype
ENSG00000106483	SFRP4	9.558	2.35E-06	0.000367	secreted frizzled related protein 4 [Source:HGNC Symbol	protein_coding
ENSG00000111057	KRT18	8.875	5.95E-12	4.38E-09	keratin 18 [Source:HGNC Symbol	protein_coding
ENSG00000170421	KRT8	8.636	3.48E-07	6.90E-05	keratin 8 [Source:HGNC Symbol	protein_coding
ENSG00000155792	DEPTOR	8.141	2.14E-09	7.58E-07	DEP domain containing MTOR interacting protein [Source:HGNC Symbol	protein_coding
ENSG00000124212	PTGIS	7.766	1.02E-06	0.000181	prostaglandin I2 synthase [Source:HGNC Symbol	protein_coding
ENSG00000181652	ATG9B	7.546	1.39E-09	5.10E-07	autophagy related 9B [Source:HGNC Symbol	protein_coding
ENSG00000111452	ADGRD1	7.364	1.81E-06	0.000316	adhesion G protein-coupled receptor D1 [Source:HGNC Symbol	protein_coding
ENSG00000115902	SLC1A4	7.12	5.41E-08	1.51E-05	solute carrier family 1 member 4 [Source:HGNC Symbol	protein_coding
ENSG00000145242	EPHA5	6.971	1.86E-05	0.0021	EPH receptor A5 [Source:HGNC Symbol	protein_coding
ENSG00000138030	KHK	6.769	6.72E-08	1.77E-05	ketohexokinase [Source:HGNC Symbol	protein_coding
ENSG00000154258	ABCA9	6.707	4.54E-07	8.67E-05	ATP binding cassette subfamily A member 9 [Source:HGNC Symbol	protein_coding
ENSG00000164619	BMPER	6.574	0.000563	0.0318	BMP binding endothelial regulator [Source:HGNC Symbol	protein_coding
ENSG00000142910	TINAGL1	6.449	0.000965	0.0452	tubulointerstitial nephritis antigen like 1 [Source:HGNC Symbol	protein_coding
ENSG00000111341	MGP	6.372	3.06E-06	0.000463	matrix Gla protein [Source:HGNC Symbol	protein_coding
ENSG00000171345	KRT19	6.37	0.000538	0.0308	keratin 19 [Source:HGNC Symbol	protein_coding
ENSG00000164107	HAND2	6.323	0.000622	0.0337	heart and neural crest derivatives expressed 2 [Source:HGNC Symbol	protein_coding
ENSG00000106484	MEST	6.156	0.000113	0.00911	mesoderm specific transcript [Source:HGNC Symbol	protein_coding
ENSG00000180914	OXTR	6.115	7.20E-06	0.00095	oxytocin receptor [Source:HGNC Symbol	protein_coding
ENSG00000163520	FBLN2	6.018	4.84E-05	0.00471	fibulin 2 [Source:HGNC Symbol	protein_coding
ENSG00000185483	ROR1	5.909	4.82E-05	0.00471	receptor tyrosine kinase like orphan receptor 1 [Source:HGNC Symbol	protein_coding
ENSG00000058085	LAMC2	5.908	0.000301	0.0202	laminin subunit gamma 2 [Source:HGNC Symbol	protein_coding

ENSG00000196557	CACNA1H	5.882	0.000363	0.0233	calcium voltage-gated channel subunit alpha1 H [Source:HGNC Symbol	protein_coding
ENSG00000106069	CHN2	5.691	0.000731	0.038	chimerin 2 [Source:HGNC Symbol	protein_coding
ENSG00000072163	LIMS2	5.682	0.000414	0.0252	LIM zinc finger domain containing 2 [Source:HGNC Symbol	protein_coding
ENSG00000070950	RAD18	5.461	0.000935	0.0448	RAD18 E3 ubiquitin protein ligase [Source:HGNC Symbol	protein_coding
ENSG00000159640	ACE	5.134	0.000194	0.0142	angiotensin I converting enzyme [Source:HGNC Symbol	protein_coding
ENSG00000169851	PCDH7	4.837	7.22E-05	0.00646	protocadherin 7 [Source:HGNC Symbol	protein_coding
ENSG00000099864	PALM	4.822	6.90E-06	0.000935	paralemmin [Source:HGNC Symbol	protein_coding
ENSG00000137809	ITGA11	4.818	1.55E-15	2.66E-12	integrin subunit alpha 11 [Source:HGNC Symbol	protein_coding
ENSG00000143994	ABHD1	4.814	0.000326	0.0216	abhydrolase domain containing 1 [Source:HGNC Symbol	protein_coding
ENSG00000204291	COL15A1	4.613	9.41E-09	2.94E-06	collagen type XV alpha 1 chain [Source:HGNC Symbol	protein_coding
ENSG00000109743	BST1	4.483	9.20E-06	0.00114	bone marrow stromal cell antigen 1 [Source:HGNC Symbol	protein_coding
ENSG00000116183	PAPPA2	4.457	8.09E-05	0.00683	pappalysin 2 [Source:HGNC Symbol	protein_coding
ENSG00000162496	DHRS3	4.438	2.18E-06	0.000351	dehydrogenase/reductase 3 [Source:HGNC Symbol	protein_coding
ENSG00000109610	SOD3	4.414	9.92E-10	3.78E-07	superoxide dismutase 3 [Source:HGNC Symbol	protein_coding
ENSG00000138131	LOXL4	4.205	3.67E-06	0.000532	lysyl oxidase like 4 [Source:HGNC Symbol	protein_coding
ENSG00000174348	PODN	4.165	9.33E-06	0.00114	podocan [Source:HGNC Symbol	protein_coding
ENSG00000076716	GPC4	4.091	0.000649	0.0348	glypican 4 [Source:HGNC Symbol	protein_coding
ENSG00000164694	FNDC1	3.971	7.77E-05	0.00667	fibronectin type III domain containing 1 [Source:HGNC Symbol	protein_coding
ENSG00000117152	RGS4	3.968	1.96E-06	0.000332	regulator of G protein signaling 4 [Source:HGNC Symbol	protein_coding
ENSG00000197614	MFAP5	3.899	1.00E-07	2.40E-05	microfibril associated protein 5 [Source:HGNC Symbol	protein_coding
ENSG00000102802	MEDAG	3.712	0.000736	0.038	mesenteric estrogen dependent adipogenesis [Source:HGNC Symbol	protein_coding
ENSG00000196517	SLC6A9	3.683	0.000302	0.0202	solute carrier family 6 member 9 [Source:HGNC Symbol	protein_coding

ENSG00000169184	MN1	3.66	1.77E-10	8.28E-08	MN1 proto-oncogene. transcriptional regulator [Source:HGNC Symbol	protein_coding
ENSG00000138061	CYP1B1	3.598	4.87E-15	7.16E-12	cytochrome P450 family 1 subfamily B member 1 [Source:HGNC Symbol	protein_coding
ENSG00000110427	KIAA1549L	3.57	0.000698	0.0368	KIAA1549 like [Source:HGNC Symbol	protein_coding
ENSG00000100395	L3MBTL2	3.45	1.84E-07	3.93E-05	L3MBTL histone methyl-lysine binding protein 2 [Source:HGNC Symbol	protein_coding
ENSG00000180044	C3orf80	3.413	0.000381	0.0239	chromosome 3 open reading frame 80 [Source:HGNC Symbol	protein_coding
ENSG00000221955	SLC12A8	3.377	0.000412	0.0252	solute carrier family 12 member 8 [Source:HGNC Symbol	protein_coding
ENSG00000178031	ADAMTSL1	3.352	4.43E-05	0.00447	ADAMTS like 1 [Source:HGNC Symbol	protein_coding
ENSG00000163453	IGFBP7	3.308	1.19E-20	6.13E-17	insulin like growth factor binding protein 7 [Source:HGNC Symbol	protein_coding
ENSG00000092621	PHGDH	3.257	3.47E-06	0.00051	phosphoglycerate dehydrogenase [Source:HGNC Symbol	protein_coding
ENSG00000109625	CPZ	3.233	0.000425	0.0256	carboxypeptidase Z [Source:HGNC Symbol	protein_coding
ENSG00000141448	GATA6	3.229	0.000284	0.0194	GATA binding protein 6 [Source:HGNC Symbol	protein_coding
ENSG00000004776	HSPB6	3.218	1.04E-10	5.64E-08	heat shock protein family B (small) member 6 [Source:HGNC Symbol	protein_coding
ENSG00000240694	PNMA2	3.197	0.000922	0.0444	PNMA family member 2 [Source:HGNC Symbol	protein_coding
ENSG00000119280	C1orf198	3.184	1.32E-05	0.00156	chromosome 1 open reading frame 198 [Source:HGNC Symbol	protein_coding
ENSG00000135069	PSAT1	3.137	3.76E-05	0.00391	phosphoserine aminotransferase 1 [Source:HGNC Symbol	protein_coding
ENSG00000151150	ANK3	3.09	0.000531	0.0307	ankyrin 3 [Source:HGNC Symbol	protein_coding
ENSG00000182492	BGN	3.03	1.20E-16	3.08E-13	biglycan [Source:HGNC Symbol	protein_coding
ENSG00000174807	CD248	3.006	1.84E-08	5.58E-06	CD248 molecule [Source:HGNC Symbol	protein_coding
ENSG00000172349	IL16	2.962	0.000738	0.038	interleukin 16 [Source:HGNC Symbol	protein_coding
ENSG00000164484	TMEM200A	2.94	0.000242	0.017	transmembrane protein 200A [Source:HGNC Symbol	protein_coding
ENSG00000206258	TNXB	2.854	1.76E-05	0.00204	tenascin XB [Source:HGNC Symbol	protein_coding
ENSG00000107731	UNC5B	2.844	4.91E-06	0.000693	unc-5 netrin receptor B [Source:HGNC Symbol	protein_coding

ENSG00000005882	PDK2	2.791	3.36E-13	3.15E-10	pyruvate dehydrogenase kinase 2 [Source:HGNC Symbol	protein_coding
ENSG00000281690	ADAMTS12	2.777	0.000814	0.0399	ADAM metallopeptidase with thrombospondin type 1 motif 12 [Source:HGNC Symbol	protein_coding
ENSG00000113083	LOX	2.756	6.00E-13	4.75E-10	lysyl oxidase [Source:HGNC Symbol	protein_coding
ENSG00000163661	PTX3	2.736	4.56E-10	1.96E-07	pentraxin 3 [Source:HGNC Symbol	protein_coding
ENSG00000138623	SEMA7A	2.632	0.000497	0.0293	semaphorin 7A (John Milton Hagen blood group) [Source:HGNC Symbol	protein_coding
ENSG00000186340	THBS2	2.602	1.22E-14	1.39E-11	thrombospondin 2 [Source:HGNC Symbol	protein_coding
ENSG00000169018	FEM1B	2.586	1.23E-07	2.87E-05	fem-1 homolog B [Source:HGNC Symbol	protein_coding
ENSG00000176658	MYO1D	2.546	5.17E-07	9.68E-05	myosin ID [Source:HGNC Symbol	protein_coding
ENSG00000115380	EFEMP1	2.507	2.33E-06	0.000367	EGF containing fibulin extracellular matrix protein 1 [Source:HGNC Symbol	protein_coding
ENSG00000120913	PDLIM2	2.498	0.000372	0.0235	PDZ and LIM domain 2 [Source:HGNC Symbol	protein_coding
ENSG00000103888	CEMIP	2.452	1.87E-07	3.93E-05	cell migration inducing hyaluronidase 1 [Source:HGNC Symbol	protein_coding
ENSG00000130635	COL5A1	2.404	2.19E-14	2.26E-11	collagen type V alpha 1 chain [Source:HGNC Symbol	protein_coding
ENSG00000108821	COL1A1	2.361	1.18E-14	1.39E-11	collagen type I alpha 1 chain [Source:HGNC Symbol	protein_coding
ENSG00000146674	IGFBP3	2.32	7.58E-06	0.000971	insulin like growth factor binding protein 3 [Source:HGNC Symbol	protein_coding
ENSG00000161544	CYGB	2.314	0.000384	0.0239	cytoglobin [Source:HGNC Symbol	protein_coding
ENSG00000047410	TPR	2.283	2.58E-06	0.000396	translocated promoter region. nuclear basket protein [Source:HGNC Symbol	protein_coding
ENSG00000170558	CDH2	2.269	4.23E-06	0.000605	cadherin 2 [Source:HGNC Symbol	protein_coding
ENSG00000101000	PROCR	2.258	0.000763	0.0387	protein C receptor [Source:HGNC Symbol	protein_coding
ENSG00000091986	CCDC80	2.245	1.98E-11	1.27E-08	coiled-coil domain containing 80 [Source:HGNC Symbol	protein_coding
ENSG00000135919	SERPINE2	2.234	1.16E-10	5.99E-08	serpin family E member 2 [Source:HGNC Symbol	protein_coding
ENSG00000162521	RBBP4	2.205	1.85E-07	3.93E-05	RB binding protein 4. chromatin remodeling factor [Source:HGNC Symbol	protein_coding

ENSG00000100234	TIMP3	2.203	2.69E-07	5.53E-05	TIMP metallopeptidase inhibitor 3 [Source:HGNC Symbol	protein_coding
ENSG00000074181	NOTCH3	2.178	7.43E-05	0.00657	notch receptor 3 [Source:HGNC Symbol	protein_coding
ENSG00000168542	COL3A1	2.172	3.94E-08	1.13E-05	collagen type III alpha 1 chain [Source:HGNC Symbol	protein_coding
ENSG00000109472	CPE	2.113	0.000785	0.0393	carboxypeptidase E [Source:HGNC Symbol	protein_coding
ENSG00000160613	PCSK7	2.1	3.95E-09	1.31E-06	proprotein convertase subtilisin/kexin type 7 [Source:HGNC Symbol	protein_coding
ENSG00000127418	FGFRL1	2.097	0.000109	0.00886	fibroblast growth factor receptor like 1 [Source:HGNC Symbol	protein_coding
ENSG00000159023	EPB41	2.088	8.36E-05	0.00699	erythrocyte membrane protein band 4.1 [Source:HGNC Symbol	protein_coding
ENSG00000105281	SLC1A5	2.075	8.37E-06	0.00105	solute carrier family 1 member 5 [Source:HGNC Symbol	protein_coding
ENSG00000174804	FZD4	2.035	7.64E-06	0.000971	frizzled class receptor 4 [Source:HGNC Symbol	protein_coding
ENSG00000150687	PRSS23	2.03	6.55E-09	2.11E-06	serine protease 23 [Source:HGNC Symbol	protein_coding
ENSG00000185112	FAM43A	1.956	0.000562	0.0318	family with sequence similarity 43 member A [Source:HGNC Symbol	protein_coding
ENSG00000087116	ADAMTS2	1.95	6.54E-05	0.00596	ADAM metallopeptidase with thrombospondin type 1 motif 2 [Source:HGNC Symbol	protein_coding
ENSG00000283802	ADAMTS2	1.95	6.54E-05	0.00596	ADAM metallopeptidase with thrombospondin type 1 motif 2 [Source:HGNC Symbol	protein_coding
ENSG00000113140	SPARC	1.82	5.49E-06	0.000763	secreted protein acidic and cysteine rich [Source:HGNC Symbol	protein_coding
ENSG00000070366	SMG6	1.764	1.99E-05	0.0022	SMG6 nonsense mediated mRNA decay factor [Source:HGNC Symbol	protein_coding
ENSG00000214140	PRCD	1.729	2.12E-06	0.000347	photoreceptor disc component [Source:HGNC Symbol	protein_coding
ENSG00000133026	MYH10	1.715	0.000327	0.0216	myosin heavy chain 10 [Source:HGNC Symbol	protein_coding
ENSG00000106366	SERPINE1	1.695	7.64E-07	0.000138	serpin family E member 1 [Source:HGNC Symbol	protein_coding
ENSG00000071967	CYBRD1	1.612	0.000807	0.0398	cytochrome b reductase 1 [Source:HGNC Symbol	protein_coding
ENSG00000164692	COL1A2	1.583	9.89E-08	2.40E-05	collagen type I alpha 2 chain [Source:HGNC Symbol	protein_coding
ENSG00000115317	HTRA2	1.514	0.000157	0.0122	HtrA serine peptidase 2 [Source:HGNC Symbol	protein_coding
ENSG00000167123	CERCAM	1.499	0.000252	0.0175	cerebral endothelial cell adhesion molecule [Source:HGNC Symbol	protein_coding

ENSG00000135502	SLC26A10	1.488	0.000159	0.0122	solute carrier family 26 member 10 [Source:HGNC Symbol	protein_coding
ENSG00000188760	TMEM198	1.481	0.000539	0.0308	transmembrane protein 198 [Source:HGNC Symbol	protein_coding
ENSG00000140545	MFGE8	1.477	2.59E-05	0.00275	milk fat globule-EGF factor 8 protein [Source:HGNC Symbol	protein_coding
ENSG00000182752	PAPPA	1.304	0.000434	0.0259	pappalysin 1 [Source:HGNC Symbol	protein_coding
ENSG00000149257	SERPINH1	1.176	0.000419	0.0254	serpin family H member 1 [Source:HGNC Symbol	protein_coding
ENSG00000164404	GDF9	1.135	0.000589	0.0326	growth differentiation factor 9 [Source:HGNC Symbol	protein_coding
ENSG00000102265	TIMP1	1.071	0.000371	0.0235	TIMP metallopeptidase inhibitor 1 [Source:HGNC Symbol	protein_coding
ENSG00000050165	DKK3	-1.205	0.00033	0.0216	dickkopf WNT signaling pathway inhibitor 3 [Source:HGNC Symbol	protein_coding
ENSG00000116016	EPAS1	-1.399	0.000787	0.0393	endothelial PAS domain protein 1 [Source:HGNC Symbol	protein_coding
ENSG00000011465	DCN	-1.44	0.000568	0.0318	decorin [Source:HGNC Symbol	protein_coding
ENSG00000152661	GJA1	-1.522	0.000965	0.0452	gap junction protein alpha 1 [Source:HGNC Symbol	protein_coding
ENSG00000164171	ITGA2	-1.626	0.000991	0.0457	integrin subunit alpha 2 [Source:HGNC Symbol	protein_coding
ENSG00000169047	IRS1	-1.631	0.000959	0.0452	insulin receptor substrate 1 [Source:HGNC Symbol	protein_coding
ENSG00000101160	CTSZ	-1.635	4.65E-05	0.00465	cathepsin Z [Source:HGNC Symbol	protein_coding
ENSG00000085662	AKR1B1	-1.728	6.89E-06	0.000935	aldo-keto reductase family 1 member B [Source:HGNC Symbol	protein_coding
ENSG00000168528	SERINC2	-1.737	0.000887	0.0429	serine incorporator 2 [Source:HGNC Symbol	protein_coding
ENSG00000141458	NPC1	-1.938	0.000565	0.0318	NPC intracellular cholesterol transporter 1 [Source:HGNC Symbol	protein_coding
ENSG00000164761	TNFRSF11B	-1.999	0.000966	0.0452	TNF receptor superfamily member 11b [Source:HGNC Symbol	protein_coding
ENSG00000106665	CLIP2	-2.037	3.00E-04	0.0202	CAP-Gly domain containing linker protein 2 [Source:HGNC Symbol	protein_coding
ENSG00000136048	DRAM1	-2.101	0.000154	0.012	DNA damage regulated autophagy modulator 1 [Source:HGNC Symbol	protein_coding
ENSG00000114251	WNT5A	-2.213	3.04E-07	6.13E-05	Wnt family member 5A [Source:HGNC Symbol	protein_coding
ENSG00000136542	GALNT5	-2.231	4.02E-05	0.0041	polypeptide N-acetylgalactosaminyltransferase 5 [Source:HGNC Symbol	protein_coding
ENSG00000065534	MYLK	-2.262	5.06E-05	0.00482	myosin light chain kinase [Source:HGNC Symbol	protein_coding

ENSG00000113851	CRBN	-2.303	0.00107	0.049	cereblon [Source:HGNC Symbol	protein_coding
ENSG00000085117	CD82	-2.329	0.000176	0.0132	CD82 molecule [Source:HGNC Symbol	protein_coding
ENSG00000115963	RND3	-2.333	1.24E-05	0.00149	Rho family GTPase 3 [Source:HGNC Symbol	protein_coding
ENSG00000222047	C10orf55	-2.402	3.74E-09	1.28E-06	chromosome 10 open reading frame 55 [Source:HGNC Symbol	protein_coding
ENSG00000135111	TBX3	-2.526	2.31E-08	6.79E-06	T-box transcription factor 3 [Source:HGNC Symbol	protein_coding
ENSG00000198743	SLC5A3	-2.609	0.000168	0.0126	solute carrier family 5 member 3 [Source:HGNC Symbol	protein_coding
ENSG00000197632	SERPINB2	-2.664	0.000274	0.0188	serpin family B member 2 [Source:HGNC Symbol	protein_coding
ENSG00000005884	ITGA3	-2.68	5.40E-07	9.93E-05	integrin subunit alpha 3 [Source:HGNC Symbol	protein_coding
ENSG00000170525	PFKFB3	-2.731	0.000205	0.0147	6-phosphofructo-2-kinase/fructose-2.6-biphosphatase 3 [Source:HGNC Symbol	protein_coding
ENSG00000148082	SHC3	-2.906	0.000987	0.0457	SHC adaptor protein 3 [Source:HGNC Symbol	protein_coding
ENSG00000101825	MXRA5	-3.065	2.08E-06	0.000346	matrix remodeling associated 5 [Source:HGNC Symbol	protein_coding
ENSG00000154736	ADAMTS5	-3.072	0.000164	0.0125	ADAM metallopeptidase with thrombospondin type 1 motif 5 [Source:HGNC Symbol	protein_coding
ENSG00000105835	NAMPT	-3.097	0.000831	0.0405	nicotinamide phosphoribosyltransferase [Source:HGNC Symbol	protein_coding
ENSG00000186469	GNG2	-3.154	0.000711	0.0374	G protein subunit gamma 2 [Source:HGNC Symbol	protein_coding
ENSG00000166260	COX11	-3.166	0.000656	0.035	cytochrome c oxidase copper chaperone COX11 [Source:HGNC Symbol	protein_coding
ENSG00000041982	TNC	-3.218	1.87E-16	3.86E-13	tenascin C [Source:HGNC Symbol	protein_coding
ENSG00000189320	FAM180A	-3.251	0.000617	0.0337	family with sequence similarity 180 member A [Source:HGNC Symbol	protein_coding
ENSG00000169429	CXCL8	-3.47	7.67E-10	3.16E-07	C-X-C motif chemokine ligand 8 [Source:HGNC Symbol	protein_coding
ENSG00000125257	ABCC4	-3.547	6.92E-05	0.00625	ATP binding cassette subfamily C member 4 [Source:HGNC Symbol	protein_coding
ENSG00000196611	MMP1	-3.572	8.51E-10	3.37E-07	matrix metallopeptidase 1 [Source:HGNC Symbol	protein_coding
ENSG00000204381	LAYN	-3.574	9.31E-05	0.00767	layilin [Source:HGNC Symbol	protein_coding

ENSG00000115461	IGFBP5	-3.781	1.36E-05	0.00159	insulin like growth factor binding protein 5 [Source:HGNC Symbol	protein_coding
ENSG00000154188	ANGPT1	-3.8	0.000761	0.0387	angiopoietin 1 [Source:HGNC Symbol	protein_coding
ENSG00000196549	MME	-3.863	1.97E-06	0.000332	membrane metalloendopeptidase [Source:HGNC Symbol	protein_coding
ENSG00000105976	MET	-4.031	1.99E-05	0.0022	MET proto-oncogene. receptor tyrosine kinase [Source:HGNC Symbol	protein_coding
ENSG00000168398	BDKRB2	-4.053	0.000641	0.0345	bradykinin receptor B2 [Source:HGNC Symbol	protein_coding
ENSG00000115756	HPCAL1	-4.129	0.000166	0.0126	hippocalcin like 1 [Source:HGNC Symbol	protein_coding
ENSG00000178177	LCORL	-4.138	0.000507	0.0295	ligand dependent nuclear receptor corepressor like [Source:HGNC Symbol	protein_coding
ENSG00000064607	SUGP2	-4.223	0.000119	0.00953	SURP and G-patch domain containing 2 [Source:HGNC Symbol	protein_coding
ENSG00000112685	EXOC2	-4.481	6.11E-05	0.00562	exocyst complex component 2 [Source:HGNC Symbol	protein_coding
ENSG00000169908	TM4SF1	-4.658	8.03E-08	2.02E-05	transmembrane 4 L six family member 1 [Source:HGNC Symbol	protein_coding
ENSG00000012817	KDM5D	-4.789	0.000368	0.0235	lysine demethylase 5D [Source:HGNC Symbol	protein_coding
ENSG00000159167	STC1	-4.805	0.000151	0.0119	stanniocalcin 1 [Source:HGNC Symbol	protein_coding
ENSG00000112282	MED23	-4.971	0.000265	0.0183	mediator complex subunit 23 [Source:HGNC Symbol	protein_coding
ENSG00000134070	IRAK2	-5.223	0.000459	0.0271	interleukin 1 receptor associated kinase 2 [Source:HGNC Symbol	protein_coding
ENSG00000164674	SYTL3	-5.225	0.00072	0.0376	synaptotagmin like 3 [Source:HGNC Symbol	protein_coding
ENSG00000178852	EFCAB13	-5.287	0.000236	0.0167	EF-hand calcium binding domain 13 [Source:HGNC Symbol	protein_coding
ENSG00000103449	SALL1	-5.312	0.000664	0.0352	spalt like transcription factor 1 [Source:HGNC Symbol	protein_coding
ENSG00000143147	GPR161	-5.452	0.000982	0.0457	G protein-coupled receptor 161 [Source:HGNC Symbol	protein_coding
ENSG00000110900	TSPAN11	-5.564	0.00108	0.0492	tetraspanin 11 [Source:HGNC Symbol	protein_coding
ENSG00000128512	DOCK4	-5.59	7.90E-05	0.00672	dedicator of cytokinesis 4 [Source:HGNC Symbol	protein_coding
ENSG00000108511	HOXB6	-5.641	0.000608	0.0335	homeobox B6 [Source:HGNC Symbol	protein_coding
ENSG00000132334	PTPRE	-5.648	0.000875	0.0425	protein tyrosine phosphatase receptor type E [Source:HGNC Symbol	protein_coding

ENSG00000139629	GALNT6	-5.649	3.88E-05	0.00399	polypeptide N-acetylgalactosaminyltransferase 6 [Source:HGNC Symbol	protein_coding
ENSG00000076706	MCAM	-5.655	0.000506	0.0295	melanoma cell adhesion molecule [Source:HGNC Symbol	protein_coding
ENSG00000160145	KALRN	-5.759	0.000576	0.0321	kalirin RhoGEF kinase [Source:HGNC Symbol	protein_coding
ENSG00000165092	ALDH1A1	-5.891	0.000946	0.0451	aldehyde dehydrogenase 1 family member A1 [Source:HGNC Symbol	protein_coding
ENSG00000101333	PLCB4	-5.897	0.000787	0.0393	phospholipase C beta 4 [Source:HGNC Symbol	protein_coding
ENSG00000181333	HEPHL1	-6.157	0.000232	0.0166	hephaestin like 1 [Source:HGNC Symbol	protein_coding
ENSG00000158270	COLEC12	-6.241	0.000622	0.0337	collectin subfamily member 12 [Source:HGNC Symbol	protein_coding
ENSG00000001617	SEMA3F	-6.353	0.000196	0.0142	semaphorin 3F [Source:HGNC Symbol	protein_coding
ENSG00000183735	TBK1	-6.562	0.000123	0.00974	TANK binding kinase 1 [Source:HGNC Symbol	protein_coding
ENSG00000187957	DNER	-6.693	2.32E-05	0.00251	delta/notch like EGF repeat containing [Source:HGNC Symbol	protein_coding
ENSG00000012171	SEMA3B	-6.701	4.84E-05	0.00471	semaphorin 3B [Source:HGNC Symbol	protein_coding
ENSG00000069020	MAST4	-6.736	3.16E-05	0.00332	microtubule associated serine/threonine kinase family member 4 [Source:HGNC Symbol	protein_coding
ENSG00000108932	SLC16A6	-6.739	3.20E-06	0.000477	solute carrier family 16 member 6 [Source:HGNC Symbol	protein_coding
ENSG00000118473	SGIP1	-6.925	7.47E-06	0.000971	SH3GL interacting endocytic adaptor 1 [Source:HGNC Symbol	protein_coding
ENSG00000149968	MMP3	-7.169	1.35E-07	3.09E-05	matrix metallopeptidase 3 [Source:HGNC Symbol	protein_coding
ENSG00000162892	IL24	-8.241	3.24E-10	1.45E-07	interleukin 24 [Source:HGNC Symbol	protein_coding
ENSG00000162894	FCMR	-8.851	4.42E-13	3.79E-10	Fc fragment of IgM receptor [Source:HGNC Symbol	protein_coding
